# Computer-based cognitive remediation program for the treatment of behavioral problems in children with intellectual disability: the «COGNITUS & MOI» study protocol for a randomized controlled trial

**DOI:** 10.1186/s12888-018-1810-z

**Published:** 2018-07-20

**Authors:** Emilie Favre, Elodie Peyroux, Marie-Noelle Babinet, Alice Poisson, Caroline Demily

**Affiliations:** 1 GenoPsy, Reference center for rare diseases with psychiatric symptoms, Centre Hospitalier le Vinatier and EDR-Psy team (CNRS UMR 5229 & Lyon 1 University), 69678 BRON Cedex Lyon, Lyon, France; 2 Faculty of Medicine “Lyon Sud Charles Meyrieux”, Lyon 1 University, Lyon, France

**Keywords:** Cognitive remediation, Intellectual disability, Children, Challenging behaviour, Comorbid psychiatric disorder

## Abstract

**Background:**

Comorbid psychiatric disorders are frequent in children with intellectual disability (ID). Given the limitations of drugs treatments, cognitive remediation could be a promising tool to reduce these challenging behaviors but evidence is still scarce. Our group recently developed the «COGNITUS & MOI» program that is designed to train the attentional and visuospatial skills in children with ID. This study investigates the efficiency of the «COGNITUS & MOI» program in this condition.

**Methods:**

Children (age: 6.00–13.11) with mild to moderate ID and behavioral problems, will benefit from a therapy during a 16 week randomized controlled trial. One group will be randomly treated with the «COGNITUS & MOI» program and the other with a motor skill and video viewing intervention. All participants will undergo a behavioral, functional and neurocognitive assessment at baseline, post-intervention, and 6-month follow-up. Primary outcome will be the change from the baseline of the score on the “hyperactivity – noncompliance” subscale of the Aberrant Behavior Checklist.

**Discussion:**

If the results are conclusive, the «COGNITUS & MOI» program could be added to the therapeutic arsenal against challenging behavior in children with ID.

**Trial registration:**

ClinicalTrials NCT02797418. Date registered: 8th of June 2016.

## Background

Intellectual disability (ID) is a neurodevelopmental medical condition defined by poor cognitive functioning and adaptive skills. It includes a lack of autonomy in daily life and weak adaptation to the environment. Comorbid psychiatric disorders frequently occur with ID [1–3]. Unfortunately, pharmacological treatments are often poorly effective and/or generate side effects [4–6]. Non-drug treatments could therefore be of particular interest. Among these approaches, cognitive remediation aims at developing strategies to overcome the daily impact of cognitive deficits [7] and could be a promising tool to reduce challenging behaviors in children with ID. Indeed, specific cognitive profiles - especially memory, attentional, visuospatial and executive deficits - are well documented in children with ID [8–10]. Impairment of these functions has been related to psychiatric and more generally to behavioral problems in other medical conditions [11, 12]. Moreover, attentional and visuospatial skills must underlie social cognition abilities, especially facial emotion recognition [13]. Impairment of social cognition frequently occurs in ID [14–16] and is associated with challenging behaviors [16, 17]. The stimulating idea that comes out of these results is that appropriate cognitive training would allow an improvement in environmental stimuli processing and consequently in the behavioral response of children with ID.

Despite these converging data, evidence-based non-drug treatments for behavioral problems in ID are still lacking. Previous studies have shown that cognitive remediation could improve cognitive abilities in children with ID [18–22] but - to our knowledge - few were interested in behavior and adaptive skills in daily life [23, 24].

Our group recently developed the «COGNITUS & MOI» program, a cognitive remediation tool designed to train the attentional and visuospatial skills of 5–13 year-old children. COGNITUS is a friendly cartoon character that accompanies the child throughout various activities in paper-and-pencil, computerized, and at-home formats [25]. The «COGNITUS & MOI» program could be of particular interest concerning challenging behaviors in children with ID for several reasons. First, the program is based on an active coaching approach and a flexible set of exercises that can be individually adapted according to the child’s clinical profile. This is especially suitable in ID because of the high heterogeneity of clinical presentation. Second, this program focuses on visuospatial and attentional skills that could be closely related to behavioral disturbances. Therefore, the focus of this randomized controlled study is the «COGNITUS & MOI» program in children with mild to moderate ID.

### Objectives of the study

The primary objective of this study is to evaluate the effectiveness of the «COGNITUS & MOI» program compared to a motor skill and video viewing intervention in terms of children’s behavioral problems.

The secondary objectives are as follows:

1. to investigate the effectiveness of the «COGNITUS & MOI» program compared to a motor skill and video viewing intervention on children’s neurocognitive abilities;
2. to investigate the effectiveness of the «COGNITUS & MOI» program compared to a motor skill and video viewing intervention on functional outcomes in children (adaptive behaviors, quality of life and self-esteem); and
3. to evaluate the long-term maintenance (6 months after) of the beneficial effects of these treatments.

It is hypothesized that children who undergo the «COGNITUS & MOI» program will demonstrate greater and more sustainable improvements in behavioral problems, neurocognitive abilities and functional outcomes than those who receive a motor skill and video viewing intervention.

## Methods and design

### Design

**Fig. 1:**
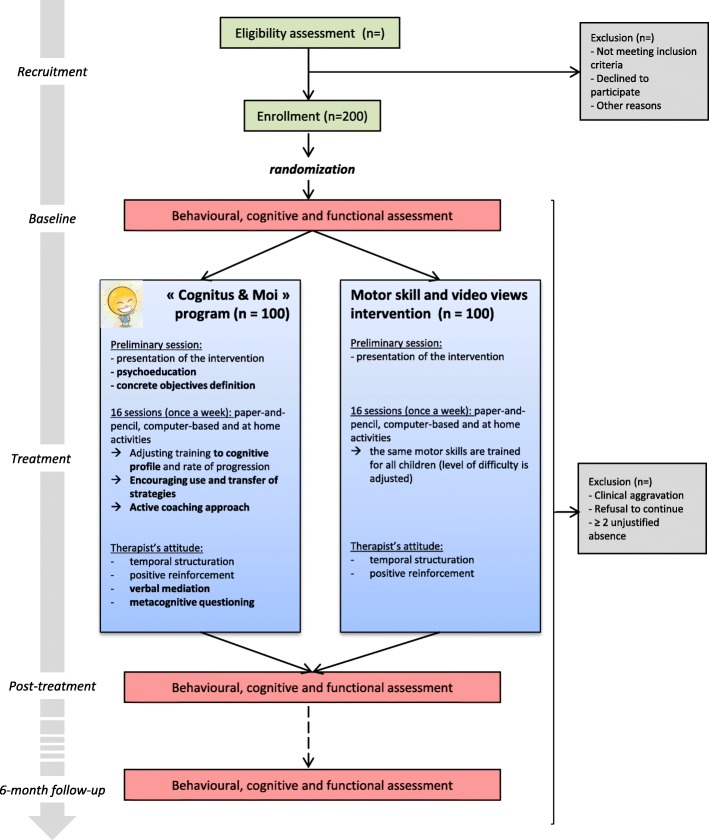
Schematic overview of the «COGNITUS & MOI» study protocol

Two groups of 100 children will benefit from a therapy during a 16 week randomized controlled trial. One group will be randomly treated with the «COGNITUS & MOI» program and the other with a motor skill and video viewing intervention. In both groups, the preliminary session will consist of the presentation of the program to the child and their parents by the therapist. The following 16 therapy sessions will occur once a week and include paper-and-pencil, computer-based and at-home activities. All participants will undergo a behavioral, functional and neurocognitive assessment by an experienced psychologist. The assessment will occur before, immediately after and 6 months after the end of the program (baseline, post-treatment and 6-month follow-up). Figure 1 shows a schematic overview of the design of the study.

### Inclusion and exclusion criteria

Children from 6 to 13 years old with mild to moderate intellectual disability in line with DSM-V criteria and behavioral problems are eligible for this study. Detailed inclusion and exclusion criteria are summarized in Table 1. Pychotropic medications and occupational, speech, orthoptic and/or psychomotor therapies should remain unchanged thorough the protocol. The intervention will be discontinued in case of clinical aggravation, patient’s refusal to continue or after more than two unjustified absence rated by therapist.

**Table 1:** Inclusion and exclusion criteria

Inclusion criteria	Exclusion criteria
• age between 6 years 0 month and 13 years 11 months • ABC scale total score equal or higher than 15 • total IQ score between 45 and 70 (WPPSI-III, WPPSI-IV, WISC-IV or WISC-V scale) AND standard score of at least one domain of the VABS-II between 40 and 70 AND measurements carried out for less than 18 months • French speaker • Psychotropic medication remained unchanged for 1 month • Occupational, speech, orthoptic and/or psychomotor therapies remained unchanged for 1 month	• DSM-5 criteria for autism spectrum disorder • vascular, infectious or degenerative neurological condition • medication for somatic problem that might interfere with brain functioning

*WPPSI* Wechsler Preschool and Primary Scale of Intelligence (Wechsler, 2004; 2014), *WISC* Wechsler Intelligence Scale for Children (Wechsler, 2005; 2016), *ABC* Aberrant Behavior Checklist (Aman & Singh, 1986), *VABS-II* Vineland Adaptive Behavior Scales – Second Edition (Sparrow, Cicchetti, & Balla, 2015)

### Recruitment

Recruitment will occur among 6 French clinical genetic, psychiatric, or neurologic departments of public hospitals. Detailed oral and written information about the study will be given to the child and their parents by the investigator. For ethical reasons, parents will be informed that children allocated to the motor skill and video viewing intervention could benefit from the «COGNITUS & MOI» program at the end of the study. Inclusion and exclusion criteria will be carefully verified by medical investigators. Parents will give written informed consent, and children will provide oral consent before inclusion in the research program.

### Randomization

Four strata will be constructed based on age (6–9 and 10–13 years) and total Intellectual Quotient (IQ) score (45–55 and 56–70) to balance characteristics between the treatment groups. After the inclusion of a child, randomization will be done by the statistician involved in the research project and provided to each therapist. The participants and their parents will rate several measures – including primary outcome measures – and will be informed of their group assignment. However, the psychologist will blindly assess the participant’s behavior, cognitive abilities and functional outcomes at baseline, post-treatment, and the 6-month follow-up.

### The «COGNITUS & MOI» program

The «COGNITUS & MOI» program is a cognitive remediation tool, specifically for children, and is dedicated to attentional and visuospatial functions. A precise description of the program has been previously published [25]. Briefly, it is based on the following four main principles:

1. targeting daily life difficulties with concrete objectives;
2. adjusting activities to the individual child’s cognitive profile and rate of progression;
3. encouraging the use and transfer of strategies while facing cognitive difficulties; and
4. a coaching approach leading to an interactive process between the therapist, child and his or her parents.

During the program, the therapist resorts to strategies or attitudes such as temporal structuration, verbal mediation, positive reinforcement and above all metacognitive questioning.

The present protocol includes 17 meetings with a therapist that has been specifically trained in the «COGNITUS & MOI» program. The first preliminary session will apply to the child and their parents. The therapist will do the following:

1. go through the behavioral, neurocognitive and functional assessment and outline the strengths and weaknesses of the child’s cognitive profile.
2. provide psychoeducation by explaining to the child and their parents what attentional and visuospatial functions are and their implications in daily life. In particular, this is achieved through a comic strip given to the child and a brochure with written information given to the parents.
3. define with the child and his parents the concrete aims of the therapy in accordance with his cognitive profile and difficulties in daily life.
4. introduce the program.
5. explain the role of at-home activities in strategy transfer and provide the child with the «COGNITUS & MOI» book (a child-friendly notebook that holds reports of at-home activities).

Each of the following 16 weekly sessions will last 1 hour and be identically structured. Each session begins with the program description and a review of the at-home activity for the previous week. The paper-and-pencil activities and computerized activities occur next and take 15 min each. The therapist creates paper-and-pencil activities on a case-by-case basis depending on the child’s cognitive profile and hobbies. The therapist chooses computerized activities from the exercises in the attentional and visuospatial modules. Each session ends with a Cognitus’ facial expression recognition task and an explanation of the at-home activity to the child and their parents.

### The motor skill and video viewing intervention

The motor skill and video viewing intervention is structured in a similar manner as the «COGNITUS & MOI» program but does not follow the same key principles. Activities are fixed in advance and are not adjusted to the child’s profile. Concrete objectives are not defined, and use of strategy is not encouraged. The therapist does not provide coaching except for positive reinforcement and temporal structuration of the sessions.

During the first session, the therapist will only explain the results of the assessment, introduce the program and provide the child with a child-friendly notebook that holds at-home activity reports.

The 16 weekly sessions will be structured in the same manner as the «COGNITUS & MOI» program except that there will be no facial expression recognition task. The therapist will adjust the paper-and-pencil activities for difficulties but will conform to a specific order with regard to the fine motor functions that are taught in each session (spreading, tying, blowing, nailing, pouring, coloring, screwing, etc.). The computerized activities consist of passive viewing of videos that are appropriate for the child’s age.

### Assessments and outcomes

Assessments will include behavioral problems, neurocognitive abilities (language, memory, praxis and visuospatial abilities and attentional and executive functions), social cognitive processes (emotion recognition and theory of mind abilities) and functional outcome (quality of life, self-esteem and adaptive and daily living skills) measures. Assessments will involve the child, their parents and an experienced psychologist.

The primary outcome is the change from the baseline of the score on the “hyperactivity – noncompliance” subscale of the Aberrant Behavior Checklist [26].

Secondary outcomes are as follows:

1. the change from the baseline of the scores for a semi-structured interview investigating adaptive behaviors and daily living skills,
2. the change from the baseline of the scores of two questionnaires investigating quality of life and self-esteem,
3. the change from the baseline of scores of paper-and-pencil subtests, computerized subtests and questionnaires investigating praxis and visuospatial functions, memory, language, attentional and executive functions, and social cognition, and
4. the change of all scores from post-treatment to the 6-month follow up.

See Table 2 for a detailed overview of the assessments and outcome measures.

**Table 2:** Measurements

	Kind of measure	Name	type	informant	recruitment	baseline assessment	post-treatment assessment	6-months follow up assessment
	IQ estimation	WPPSI-III, WPPSI-IV, WISC-IV or WISC-V	P&P	C	*			
*primary outcome*	behavioural problems	ABC	Q	P	*	*	*	*
*secondary outcome*	adaptive and daily living skills	VABS-II	S Int.	P	*	*	*	*
	quality of life	KIDSCREEN-27	Q	P		*	*	*
	self esteem	self-esteem subscale MDI-C	Q	C		*	*	*
	lexical knowledge	PPVT	P&P	C		*	*	*
	learning and long term memory	word list CMS	P&P	C		*	*	*
		dot locations CMS	P&P	C		*	*	*
	praxis and visuospatial functions	visuomotor precision NEPSY-II	P&P	C		*	*	*
		imitating hand positions NEPSY-II	P&P	C		*	*	*
		arrows NEPSY-II	P&P	C		*	*	*
		block construction NEPSY-II	P&P	C		*	*	*
		route finding NEPSY-II	P&P	C		*	*	*
	attentional functions	sky search TEA-Ch	P&P	C		*	*	*
		alertness TAP 2.3	CT	C		*	*	*
		auditory attention NEPSY-II	P&P	C		*	*	*
		divided attention TAP 2.3	CT	C		*	*	*
	executive functions	Go/NoGo TAP 2.3	CT	C		*	*	*
		response set NEPSY-II	P&P	C		*	*	*
		inhibition NEPSY-II	P&P	C		*	*	*
		mazes WISC-III	P&P	C		*	*	*
	social cognition	affect recognition NEPSY-II	P&P	C		*	*	*
		theory of mind NEPSY-II	P&P	C		*	*	*
		EQS	Q	P		*	*	*

*WPPSI* Wechsler Preschool and Primary Scale of Intelligence (Wechsler, 2004; 2014), *WISC* Wechsler Intelligence Scale for Children (Wechsler, 2005; 2016), *ABC* Aberrant Behavior Checklist (Aman & Singh, 1986), *VABS-II* Vineland Adaptive Behavior Scales – Second Edition (Sparrow, Cicchetti, & Balla, 2015), *KIDSCREEN-27* (Robitail et al., 2007), *MDI-C* multiscore Depression Inventory for Children (Berndt & Kaiser, 1999), *PPVT* Peabody Picture Vocabulary Test (Dunn, Thériault-Whalen, & Dunn, 1993), CMS Children’s Memory Scale (Cohen, 2001), *NEPSY-II* Developmental NEuroPSYchological Assessment – Second Edition (Korkman, Kirk, & Kemp, 2012), *TEA-Ch* Test of Everyday Attention for Children (Manly, Robertson, Anderson, & Mimmo-Smith, 2004), *TAP 2.3*. Test of Attentional Performance – Version 2.3 (Zimmermann & Fimm, 2012), *WISC-III* Wechsler Intelligence Scale for Children - Third Edition (Wechsler, 1996), *EQS* Empathy Quotient Questionnaire (Baron-Cohen & Wheelwright, 2004); *P&P* paper and pencil test; *Q* questionnaire; *S* Int. semi structured interview; *CT* computerized test; *C* child; *P* parents

### Sample size

To our knowledge, only one previous study has evaluated the effectiveness of a non-drug treatment using the “hyperactivity – noncompliance” score of the ABC as the primary outcome measurement [27]. A decrease of 3 points after treatment was found to be significant (M = 12.1; SD = 8.9 versus M = 9; SD = 7.4). Therefore, we hypothesize that following the motor skills and video viewing intervention, we will observe a 3 point decrease of the “hyperactivity – noncompliance” score. We expect that the decrease will be double for the «COGNITUS & MOI» program: the difference between the two treatments would be at least 3 points. With an expected SD of 5, a power of 90%, 1:1 allocation into the two groups, two-sided testing and a significance level of 5%, a total sample of 126 participants is required. Given the insufficient information from the literature and the risk of dropout, 200 participants will be enrolled in the present study.

### Statistical analysis

Descriptive statistics will be reported for each variable of interest for the two groups.

First, independent-sample t-tests or non-parametric Mann-Whitney tests will be run to assess the lack of differences between groups in age, IQ or severity of ABC scores at baseline (*p* < 0.05, two-sided).

Second, independent-sample t-tests or non-parametric Mann-Whitney tests will be run to compare the “hyperactivity – noncompliance” score change from the baseline to post-treatment assessment in the two groups (*p* < 0.05, two-sided).

Third, similar tests will be run for each secondary outcome measure. The score changes will be separately considered from the baseline to post-treatment assessment and from the post-treatment to 6-month follow-up assessment (*p* < 0.05, two-sided).

## Discussion

Drug treatments are often unsatisfactory methods to overcome challenging behaviors in children with mild to moderate ID. Cognitive remediation could be a promising alternative. To our knowledge, no program has been designed and validated for this clinical population. It is thus of great importance to undertake a treatment-efficacy study. The «COGNITUS & MOI» study specifically investigates the efficiency of a cognitive remediation program designed for children with mild to moderate ID.

The primary strength of this study is the «COGNITUS & MOI» program itself. It is of particular interest in ID considering that these patients have heterogeneous cognitive profiles, have few adaptive skills in daily life, and often feel incompetent. The «COGNITUS & MOI» program allows for the precise adaptation to individual clinical and cognitive profiles. Furthermore, it is based on active coaching that favors positive reinforcement and metacognitive training. It aims at improving daily life by encouraging strategy transfer. The partnership with the parents must also be emphasized because it allows for the consideration of the context of the child’s family unit. The randomized design is another strong point of the present study, highlighting the specific effect of the «COGNITUS & MOI» program versus a nonspecific intervention. It is well-known that ‘therapeutic alliance’ explains a significant proportion of any behavioral therapy efficiency. Therefore, it is very important to differentiate these general effects to validate the «COGNITUS & MOI» cognitive remediation program. The evaluation of the long-term maintenance of beneficial effects is also of particular relevance.

One limitation of this study may be that the primary outcome is assessed in an open-label fashion. Parents score their children using the ABC scale and know the intervention their child received. The use of this kind of measure is nevertheless fully justified by the fact that the study takes into account functional outcomes in everyday life, and parents receive standardized instructions to limit potential biases. Another limitation could concern the feasibility of the cognitive remediation program in children with ID. For example, it would be fair to question the use of metacognitive questioning with these children and their attendance throughout the program. Preliminary case studies confirm that the «COGNITUS & MOI» program is fully adapted to this clinical population, even in the case of poor verbal expression abilities.

## Conclusion

If the results are conclusive, the «COGNITUS & MOI» program could be added to the therapeutic arsenal against challenging behavior in children with ID. This non-drug treatment would be of particular interest because of the minimal adverse effects, which are present with drug treatments. It might also be a favorable therapy throughout life because cognitive stimulation in children could help achieve developmental milestones. Another possibility is the evaluation of the «COGNITUS & MOI» program in improving behavior and adaptive skills in adults with moderate ID, for which there are currently few adequate behavioral therapies.

## Abbreviations

ABC: Aberrant Behavior Checklist
ID: Intellectual Disability
IQ: Intellectual Quotient
